# Antibacterial Modification of Kirschner Wires with Polyluteolin toward Methicillin-Resistant *Staphylococcus aureus* (MRSA)

**DOI:** 10.3390/ma8084876

**Published:** 2015-07-30

**Authors:** Jialiang Zhu, Yantao Zhao, Lin Yang, Shuxun Hou, Yanli Su, Rungong Yang

**Affiliations:** 1 Department of Orthopedics, the First Affiliated Hospital of the General Hospital of Chinese People’s Liberation Army, Beijing 100048, China; E-Mails: orthozack@gmail.com (J.Z.); zhaoyt1980@gmail.com (Y.Z.); yanglin1988304@gmail.com (L.Y.); orthoxfcj@gmail.com (S.H.); 2 Department of Infection and Critical Care Medicine, Beijing Friendship Hospital, Capital Medical University, Beijing 100050, China

**Keywords:** Kirschner wires, antibacterial coatings, polyluteolin, steam sterilization, MRSA

## Abstract

In this study we report antibacterial modification of Kirschner wires (K-wires) with polyluteolin (PL) toward methicillin-resistant Staphylococcus aureus (MRSA). K-wires were modified by immersing them in the luteolin-containing aqueous solution for 24 h. Characterizations using scanning electron microscopy and electrochemical methods confirmed the presence of the PL coatings on the K-wires. The PL-coated K-wires were further found to show antibacterial activity toward MRSA and remained unimpaired antibacterial activity even after the steam sterilization treatment.

## 1. Introduction

Orthopaedic devices used in joint and hip replacement surgeries have greatly improved the health outcomes for patients. However, implant associated infections remain a major problem in hospital settings [1,2]. Treatment of the open fracture exposes the sterile body sites to the external environment that leads to introduction of the pathogen either on the implant itself or into the surgical site [3]. For instance, in orthopaedics periprosthetic infections have been observed to occur with a frequency of 1.5%–2.5% in primary hip and knee arthroplasties and with a frequency of 3.2%–5.6% in the revision surgery [4]. Consequences of implant-associated infections include severe osteomyelitis, prolonged hospitalization with systemic antibiotic therapy, several revision procedures, possible amputation, and even death. Gram-positive organisms, particularly staphylococcus, are responsible for the majority of implant-associated infections [5]. Among the staphylococcal species, Staphylococcus aureus and Staphylococcus epidermidis are respectively at the first and the second positions in the list of the leading etiologic agents [6]. To make matter worse, in S. aureus and S. epidermidis, the bacteria resistant to β-lactams, especially those belonging to the penicillin group, are nowadays extremely widespread. It has been reported that in isolates from orthopedic infections associated to biomaterials, about four out of five strains do not respond any longer to penicillin drugs, e.g., cephalosporins, meanwhile approximately four out of ten show methicillin/oxacillin resistance [7]. This makes the empherical therapy of implant infections, particularly those infected with methicillin-resistant Staphylococcus aureus (MRSA), a bigger challenge with limited choice of antibiotics. Therefore, there is a keen interest in developing antibacterial biomaterials to prevent medical device-associated infections. Kirschner wire or K-wires are sharpened pins, introduced in 1909 by Martin Kirschner [8]. K-wires are widely used in orthopaedics and other types of medical and veterinary surgery. They are used to hold bone fragments together (pin fixation) or to provide an anchor forskeletal traction. Because K-wires often pass through the skin into bone, they form a potential passage for bacteria from the skin to migrate into the bone and cause an infection. Therefore, it is highly desirable to develop an antibacterial coating on the K-wires.

**Figure 1:**
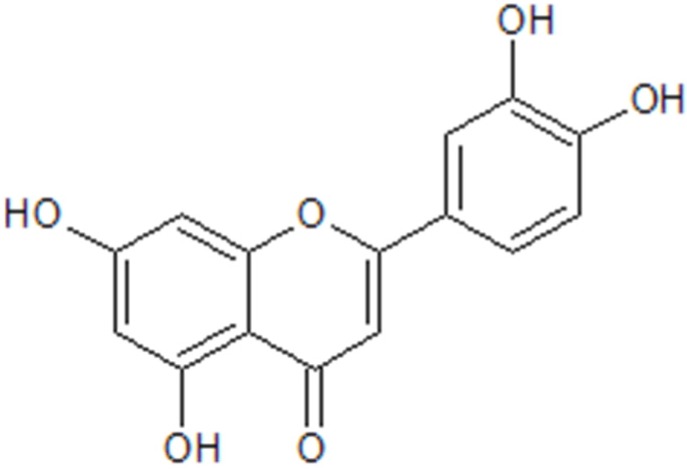
Molecular structure of luteolin.

Flavonoids belong to an important group of naturally occurring phenol derivatives. Flavonoids from fruits, vegetables and cereals, herbs and spices have shown beneficial effects on human health, and have been found to be effective antimicrobial substances against a wide variety of microorganisms [9,10,11]. Chemically they are C6-C3-C6 compounds. Luteolin (see Figure 1) belongs to the flavones subclass of the naturally occurring flavonoids. Recent studies have revealed that luteolin possesses various biological effects, such as antioxidant, anti-inflammatory and anticancer properties as well as antibacterial activity against a number of bacteria [12,13,14]. In addition, importantly, luteolin has recently been found to possess antibacterial activity toward methicillin-sensitive Staphylococcus aureus (MSSA) and MRSA [15]. On the other hand, luteolin has inhibitory activities toward both osteoclast differentiation and functions through inhibition of receptor activator of nuclear factor-κB ligand (RANKL)-induced signaling pathway as well as actin ring disruption, respectively [16]. Interestingly, more recently, luteolin-derived coatings formed by self-polymerization of luteolin have been reported to possess substrate-independent deposition and antibacterial properties [17]. In the present study, we report the antibacterial functionalization of the K-wires with polyluteolin (PL). K-wires were modified with PL just by immersing them in the luteolin-containing solution. The formation of PL coatings on the K-wires was examined with scanning electron microscopy (SEM) and electrochemical methods. Then, antibacterial activity of the PL-coated K-wires was tested with MRSA.

## 2. Results and Discussion

### 2.1. Characterization of PL-Coated K-Wires

**Figure 2:**
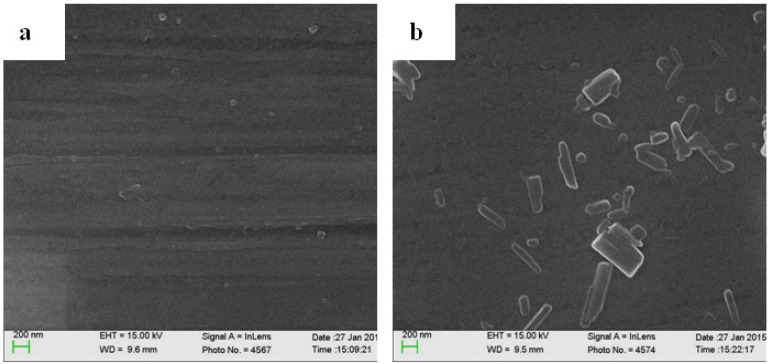
SEM images of bare Kirschner wires (K-wires) (**a**) and the polyluteolin (PL)-coated K-wires (**b**).

PL-coated K-wires could be prepared by the simple immersion of the K-wires in a freshly prepared aqueous luteolin solution for 24 h, meanwhile the color of luteolin solution changed from the initial colorless to the light yellow, indicative of the spontaneous polymerization of luteolin by molecular oxygen. PL-coated K-wires were firstly examined by SEM. As revealed by Figure 2, bare K-wires showed characteristic stripes (Figure 2a); while such features of the K-wires disappeared at the PL-coated K-wires (Figure 2b) and some aggregates associated with the PL polymers could be seen, indicating the presence of the PL coatings on the K-wires.

**Figure 3:**
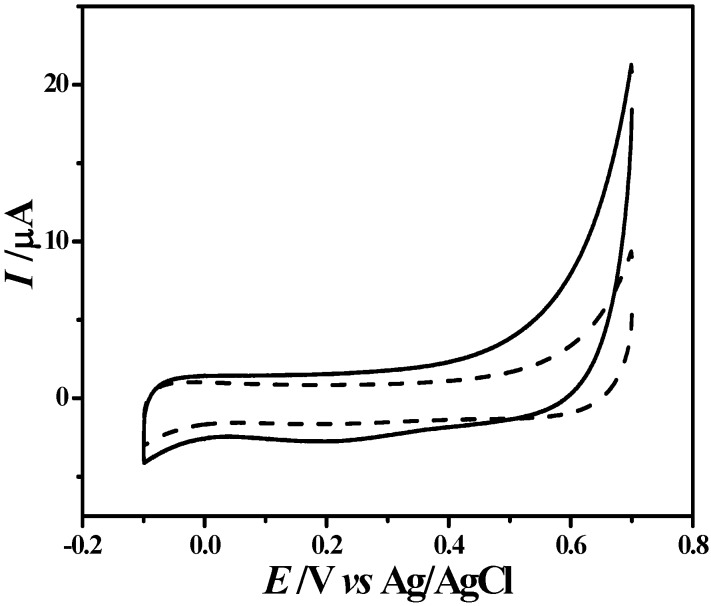
Cyclic voltammetric curve of the PL-coated K-wire (solid line) in the blank phosphate buffer solution (0.1 M, pH 7.0). As a comparison, cyclic voltammetric curve of bare K-wire was also presented (dashed line). Scan rate: 0.1 V s^−1^.

Then, cyclic voltammetry was further used to check the presence of PL coatings on the K-wires due to the redox properties associated with the PL coatings. Figure 3 shows cyclic voltammetric responses of the K-wires as electrode before and after PL coating in a blank electrolyte solution (0.1 M PBS, pH 7.0). Before PL coating, the bare K-wires electrode exhibited a typical charging/discharging current curve without any faradic process (dashed line). After PL coating, an obvious oxidation current (solid line) could be observed at potentials positive to +0.2 V *vs.* Ag/AgCl, indicating the formation of the PL coatings. The observed oxidation current could result from the oxidation of the catechol group on the ring B of luteolin structure and the irreversible oxidation of the resorcinol group on the ring A of luteolin structure [18]. During the reverse wave scans, there was a small single reduction peak at +0.2 V. This reduction peak could be a result of the reduction of the oxidation product of the catechol groups on the ring B. This phenomenon has been reported previously [19,20]. These results indicate clearly the presence of the PL coatings on the K-wires.

### 2.2. Characterization of In Vitro Antibacterial Activity of PL-Coated K-Wires

**Figure 4:**
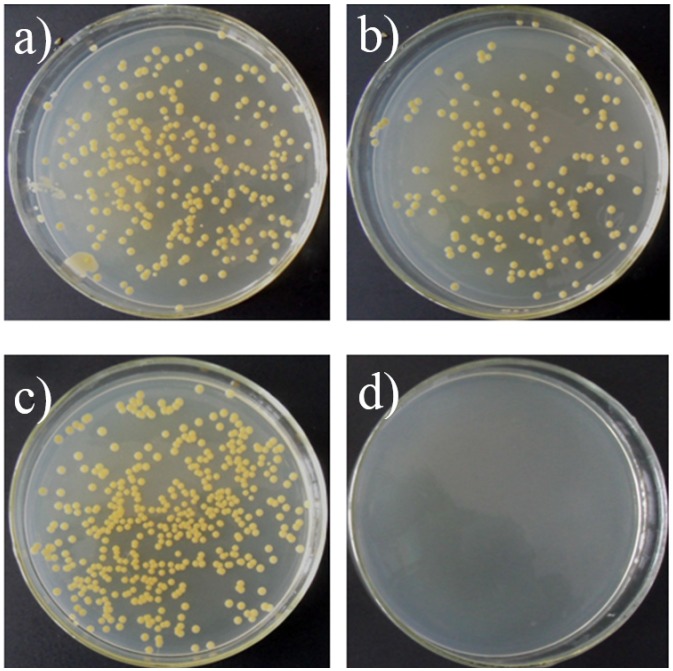
Typical images of the colonies grown on the agar plate inoculated with the methicillin-resistant *Staphylococcus aureus* (MRSA) suspension after treated with the bare K-wires (**a** and **b**) and the PL-coated K-wires (**c** and **d**) for 1 h (**a**,**c**) and 24 h (**b**,**d**).

The antibacterial activity of the PL-coated K-wires against MRSA was evaluated by ASTM E2149-01, a quantitative antibacterial test method for determining the antibacterial activity of the attached antibacterial agents under dynamic contact conditions. As shown in Figure 4, after 24 h incubation, no obvious decrease in the bacterial colonies on the agar plate inoculated with the bacterial suspension which were treated with the bare K-wires, however, no colonies were formed on the agar plate inoculated with the bacterial suspension which were treated with the PL-coated K-wires, indicating the antibacterial ability of the PL-coated K-wires.

### 2.3. Antibacterial Stability

It is well known that all materials implanted within the body or placed in contact with corporeal fluids must be sterilized and steam sterilization is the preferred sterilization process [21]. However, the steam sterilization might impair the performances of polymeric materials, such as chitosan films [22]. Therefore, the effect of steam sterilization treatments on the antibacterial activity of the PL-coated K-wires against MRSA was examined. The PL-coated K-wires were subjected to steam sterilization at 121 °C for 30 min and their antibacterial activity was evaluated. It was found that the bactericidal ratio of the PL-coated K-wires was 99.8%. Then, steam sterilization and the subsequent incubation process were repeated again. It was found that the bactericidal ratio of the PL-coated K-wires was 99.4%. These results indicate that the PL-coated K-wires could exhibit unaffected antibacterial activity even after high temperature steam sterilization. Such unimpaired antibacterial activity by steam sterilization treatment provides the PL-coated K-wires with extra advantages for practical applications.

## 3. Experimental Section

### 3.1. Modification of K-Wires with PL Coatings

K-wires (316L stainless steel alloy, length: 1 cm, diameter: 0.6 mm, Synthes. Monument, CO, USA) were immersed in a freshly prepared Tris buffer (25 mM, pH 8.5) solution containing 0.10 mM luteolin (Nanjing tcm Institute of Chinese Materia Medica, Nanjing, China) for 24 h at 37 °C.

### 3.2. Characterization of the PL-Coated K-Wires

The morphology of the PL-coated K-wires was characterized using a ZEISS Supra 55 FE-SEM. Cyclic voltammetry was used to check the presence of PL coatings on the K-wires. Cyclic voltammetric measurements were performed with a CHI 660D electrochemical analyzer (CH Instruments, Shanghai, China) with a three-electrode configuration. K-wires were used as the working electrode, Ag/AgCl (KCl-saturated) electrode as the reference electrode, and platinum coil as the counter electrode.

### 3.3. In Vitro Antimicrobial Activity

The antimicrobial properties of the PL-coated K-wires were evaluated by ASTM E2149-01, which is a quantitative antimicrobial test method performed under dynamic contact conditions. Each specimen was sterilized before the experiment with high-pressure steam sterilizer (YX-280D, Jiangyin binjiang Medical Equipment Co., Ltd, Jiangyin, China) at 121 °C for 30 min. Gram-positive bacteria, MRSA ATCC43300, was used as test organism. Strains were stored frozen in glycerol broth at −70 °C and subcultured to ensure purity before testing.

A colony of MRSA grown on a LB agar plate was cultivated in sterilized LB broth and then incubated overnight at 37 °C with a shaking incubator. Then, bacteria were suspended in 1.5 mL culture broth (10^5^ colony forming units (CFU) per mL) and incubated with the PL-coated K-wires at 225 rpm on a shaker for 1 and 24 h. At the end of the incubation, 100 μL solution was took out and plated onto 10-cm LB culture medium plates overnight, respectively. After 24 h incubation, the number of colonies on each plate was quantitated following protocols set forth by the U.S. Food and Drug Administration in their Bacteriological Analytical Manual-Aerobic Plate Count Method [23]. Briefly, the bacteria collected in 100 μL solution were diluted as necessary and plated onto the plates for the quantitation. If the resultant colonies per plate were within 25–250, the undiluted colony numbers were utilized for quantitation. If there were over 250 colonies per plate, the bacterial solution was diluted by factors of 10 (e.g., 1:10, 1:100, 1:1000 dilutions) until resultant colonies per plate were again within 25–250, and colony numbers were then calculated accordingly. An untreated K-wire was used as a control. All determinations were made in triplicate.

### 3.4. Antibacterial Stability Test

For testing the effect of steam sterilization on the antimicrobial activity of the PL coatings, the PL-coated K-wires were treated with high-pressure steam sterilizer at 121 °C for 30 min, and then were incubated with 1.5 mL of cell suspension in a 2 mL conical tube at 37 °C with a shaking incubator. This steam sterilization and the subsequent incubation processes were repeated twice. Samples were taken out after 24 h, and plated on LB agar plates. The inoculated plates were incubated at 37 °C for 24 h, and the surviving cells were counted. All determinations were made in triplicate. The antimicrobial activity was expressed as R = % reduction of the organism after contact with the test samples compared to the number of S. aureus cells surviving after contact with the control.

## 4. Conclusions

In the present study, the K-wires have been modified with the PL for producing antibacterial activity toward MRSA. Modification of the K-wires with PL coatings was facile just by immersing them in the luteolin-containing aqueous solution for 24 h. Characterization with SEM and cyclic voltammetry indicated the presence of the PL coatings on the K-wires. The PL-coated K-wires were found to possess good antibacterial activity toward MRSA. Moreover, such antibacterial activity was unimpaired by the steam sterilization, which will facilitate the practical application of the PL-coated K-wires.

## References

[1] Widmer A.F. (2001). New developments in diagnosis and treatment of infection in orthopaedic implants. Clin. Infect. Dis..

[2] Goodman S.B., Yao Z., Keeney M., Yang F. (2013). The future of biologic coatings for orthopaedic implants. Biomaterials.

[3] McMillan D.J., Lutton C., Rosenzweig N., Sriprakash K.S., Goss B., Stemberger M., Schuetz M.A., Steck R. (2011). Prevention of Staphylococcus aureus biofilm formation on metallic surgical implants via controlled release of gentamicin. J. Biomed. Sci. Eng..

[4] Montanaro L., Speziale P., Campoccia D., Ravaioli S., Cangini I., Pietrocola G., Sandro Giannini S., Arciola C.R. (2011). Scenery of Staphylococcus implant infections in orthopedics. Future Microbiol..

[5] Arciola C.R., An Y.H., Campoccia D., Donati M.E., Montanaro L. (2005). Etiology of implant orthopedic infections: A survey on 1027 clinical isolates. Int. J. Artif. Organs..

[6] Von Eiff C., Arciola C.R., Montanaro L., Becker K., Campoccia D. (2006). Emerging Staphylococcus species as new pathogens in implant infections. Int. J. Artif. Organs..

[7] Campoccia D., Montanaro L., Arciola C.R. (2006). The significance of infection related to orthopedic devices and issues of antibiotic resistance. Biomaterials.

[8] Franssen B.B.G.M., Schuurman A.H., Van Der Molen A.M., Kon M. (2010). One century of Kirschner wires and Kirschner wire insertion techniques: A historical review. Acta Orthop. Belg..

[9] Cushnie T.P.T., Lamb A.J. (2011). Recent advances in understanding the antibacterial properties of flavonoids. Int. J. Antimicrob. Agents.

[10] Abreu A.C., McBain A.J., Simoes M. (2012). Plants as sources of new antimicrobials and resistance-modifying agents. Nat. Prod. Rep..

[11] Cushnie T.P.T., Lamb A.J. (2005). Antimicrobial activity of flavonoids. Int. J. Antimicrob. Agents.

[12] Lv P.C., Li H.Q., Xue J.Y., Shi L., Zhu H.L. (2009). Synthesis and biological evaluation of novel luteolin derivatives as antibacterial agents. Eur. J. Med. Chem..

[13] Yamamoto H., Ogawa T. (2002). Antimicrobial activity of perilla seed polyphenols against oral pathogenic bacteria. Biosci. Biotechnol. Biochem..

[14] Yao J., Zhang Q., Min J., He J., Yu Z. (2010). Novel enoyl—ACP reductase (FabI) potential inhibitors of Escherichia coli from Chinese medicine monomers. Bioorg. Med. Chem. Lett..

[15] Su Y., Ma L., Wen Y., Wang H., Zhang S. (2014). Studies of the in vitro antibacterial activities of several polyphenols against clinical isolates of methicillin-resistant Staphylococcus aureus. Molecules.

[16] Lee J.W., Ahn J.Y., Hasegawa S., Cha B.Y., Yonezawa T., Nagai K., Seo H.J., Jeon W.B., Woo J.T. (2009). Inhibitory effect of luteolin on osteoclast differentiation and function. Cytotechnology.

[17] Cheng J.Y., Guan M., Zhu J.L., Wang C.T., Su L., Zhang X.J. (2014). Facile and material-independent fabrication of poly(luteolin) coatings and their unimpaired antibacterial activity against Staphylococcus aureus after steam sterilization treatments. Polym. Chem..

[18] Vestergaard M., Kerman K., Tamiya E. (2005). An electrochemical approach for detecting copper-chelating properties of flavonoids using disposable pencil graphite electrodes: Possible implications in copper-mediated illnesses. Anal. Chim. Acta.

[19] Brett A.M.O., Ghica M.E. (2003). Electrochemical oxidation of quercetin. Electroanalysis.

[20] Janeiro P., Brett A.M.O. (2004). Catechin electrochemical oxidation mechanisms. Anal. Chim. Acta.

[21] Marreco P.R., da Luz Moreira P., Genari S.C., Moraes A.M.J. (2004). Effects of different sterilization methods on the morphology, mechanical properties, and cytotoxicity of chitosan membranes used as wound dressings. Biomed. Mater. Res. Part B.

[22] Rao S.B., Sharma C.P. (1995). Sterilization of Chitosan: Implications. J. Biomater. Appl..

[23] Liu Y., Zheng Z., Zara J.N., Hsu C., Soofer D.E., Lee K.S., Siu R.K., Miller L.S., Zhang X., Carpenter D. (2012). The antimicrobial and osteoinductive properties of silver nanoparticle/poly (DL-lactic-co-glycolic acid)-coatedstainless steel. Biomaterials.

